# Graphene oxide-based electrochemical activation of ethionamide towards enhanced biological activity†

**DOI:** 10.1039/c9ra06681k

**Published:** 2019-11-01

**Authors:** Balaji B. Mulik, Sambhaji T. Dhumal, Vijay S. Sapner, Naziya N. M. A. Rehman, Prashant P. Dixit, Bhaskar R. Sathe

**Affiliations:** Department of Chemistry, Dr Babasaheb Ambedkar Marathwada University Aurangabad 431004 Maharashtra India bhaskarsathe@gmail.com; Department of Microbiology, Dr Babasaheb Ambedkar Marathwada University Aurangabad, Sub-Campus Osmanabad MH 413501 India

## Abstract

The electrochemical behavior of ethionamide (ETO) was investigated on GO (∼500 nm) using the linear sweep voltammetric (LSV) technique at the sweep rate of 10 mV s^−1^ in 1 M PBS buffer solution, and the characteristic anodic signal was examined at 0.240 V over the potential range of −0.4 to 1 V *vs.* SCE. However, linearity was observed with the increase in scan rate (2–300 mV s^−1^) and concentration of ETO (1 μM to 100 mM), suggesting that the process involved diffusion-controlled electron transfer. The results also exhibited excellent current and potential stability, limit of detection (LOD 1.33) and limit of quantification (LOQ 4.4) at optimized experimental conditions. This electrochemical oxidation method was successfully applied in the complete oxidation of ETO to its oxidized form, which was further confirmed by high resolution mass spectroscopy (HRMS) and Fourier transform infrared (FTIR) spectroscopic measurements. Interestingly, the comparative biological evaluation of ETO and ETO-O (oxidised form) showed good enhancement in the activity of oxidised ETO against some Gram-negative pathogens, such as *E. aerogenes*, *S. abony*, *S. boydii*, and *E. coli*.

## Abstract 2

Electrochemical oxidative activation of ethionamide (ETO) on GO (∼500 nm) confirmed by HRMS and FTIR analysis. The ETO-O (oxidized form) showed enhancement in activity over ETO against Gram negative pathogens (*E. aerogenes*, *S. abony*, *S. boydii*, *E. coli*).

## Graphical abstract

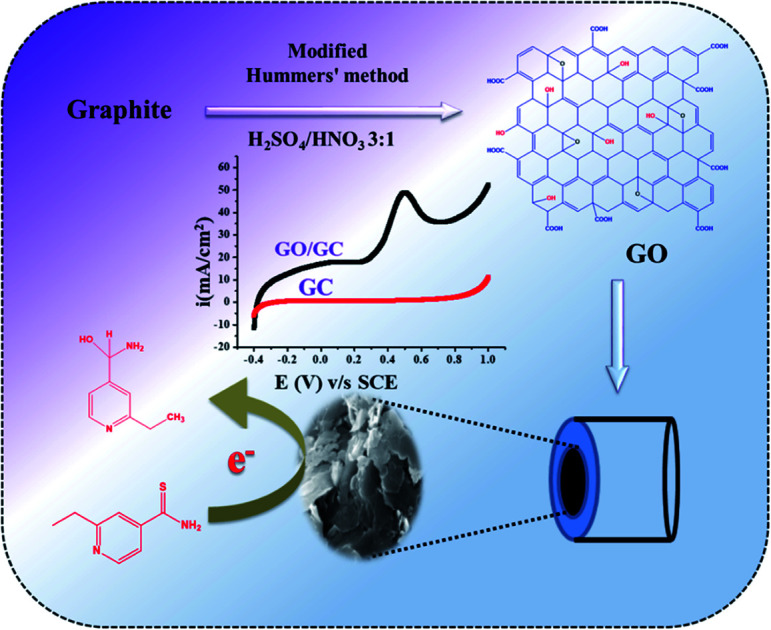

## 1. Introduction

Tuberculosis (TB) is a highly infectious disease caused by the pathogen *Mycobacterium tuberculosis* (MTB).^1^ TB mainly targets lungs besides all other parts of the human body.^2^ According to the World Health Organization (WHO), one-third of the world's population has been infected by TB, leading to 1.6 million deaths, and 10 million people were diagnosed by the end of 2018.^3^ The population of patients having Human Immunodeficiency Virus (HIV) with TB is the main cause of death from a single infectious disease.^4^ Nowadays, DOTS (directly observed treatment, short term), first-line and second-line multi-component patterns of treatments are being practiced; however, they are facing serious threats. For example, DOTS therapy needs a longer duration for treatment, and therefore, these pathogenic strains acquire resistance to the drugs.^5^

Ethionamide (ETO) or 2-ethyl thiosonicotinamide is a second line anti-tubercular drug used for treating TB. It is a structural analog of ionizing and bacteriostatic in nature^6^ and has been commonly used for the treatment of multi-drug resistant tuberculosis (MDR-TB). ETO is generally administered every 2 to 3 h repeatedly due to its half-life.^7^ Even though ETO is an effective drug, it causes some serious side effects, including hypotoxicity, gastrointestinal disorders, cardiovascular effects and neurotoxicity. Since ETO is mostly metabolised in the liver, approximately 5 to 7% of the unchanged drug is excreted through urine, while comparatively a high dose of the antibiotic remains in the body, which results in the side effects.^8^
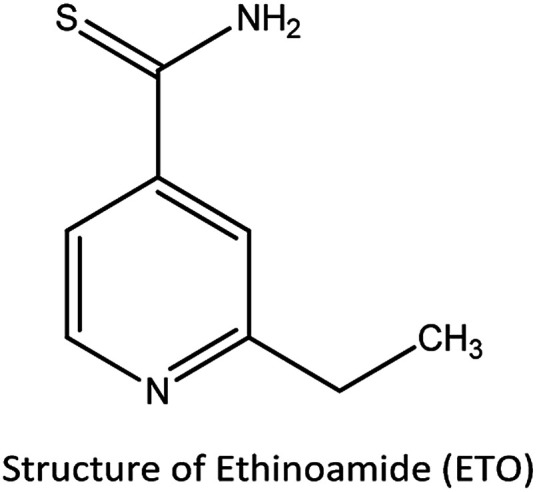


Considering the above mentioned side effects, researchers have developed various analytical tools for the determination and quantification of ETO from various formulations and even real samples.^9–15^ For example, Sujitkumar *et al.* reported a UV-Vis spectrophotometric method for ETO estimation from bulk and tablets.^10^ Madni *et al.* gave an account of an HPLC method for the determination of ETO from serum.^11^ Moreover, the spectrometric estimation of ETO was carried out using Folin–Ciocalteu reagent with iron(iii)-ferricyanide as the chromogenic agent complexing with ETO.^13^ Volumetric determination of ETO in pharmaceutical formulations was carried out by using cerium(iv) as the oxidimetric agent.^14^ These aforementioned methods had certain limitations, such as volumetric constraint, low sensitivity, usage of toxic chemicals, HPLC based methods requiring filtration and degassing, labor-intensiveness, and need of expertise for handling, longer process time and high cost of reagents. Compared to all the above, electrochemical methods have shown high sensitivity and are easy to handle and selective.^15^ Significantly, earlier reports have examined numerous electro-analytical procedures. Jain and his group reported ETO quantification using zirconia nanoparticles, whereas Bruno Ferraz reported a boron-doped diamond electrode, and Bruno Regis and coworkers determined ETO and pyrazinamide (PZA) simultaneously from pharmaceutical and urine samples by using poly l-cysteine modified electrodes.^16–18^ Unfortunately, in all the above methods, the electrode systems exhibited serious limitations, including the use of toxic or high-cost metals, low sensitivity and selectivity, and toxic chemicals.

Moreover, the low sensitivity and selectivity of the unmodified electrodes could be due to the availability of less number of active sites (surface area) and poor reproducibility. Among known electrode materials, carbon-based materials have shown high activity, stability, mechanical strength, and ease of metal atom doping, due to which they have attracted wide research interest in several fields.^19^ Among the existing carbon allotropic forms, graphene oxide (GO) has one of the efficient nanostructures, which enables significantly high mobility for electron transfer and presents a large surface area, low weight and high mechanical strength, making it an excellent electrocatalyst over the parent form *i.e.* graphite.^20^ Because its 2D structure is separated by a single layer of carbon atoms, it exhibits a hexagonal structure, which enhances the conductivity, thickness, and mechanical strength along with high surface area, and these layers are separated in aqueous solvents, which is an important criterion for the bio-compatibility of an electrocatalyst for the determination of pharmaceutical samples.^21,22^ Moreover, the electrocatalytic activity of an electrocatalyst is considerably governed by the properties of the nanomaterials, from which the catalysts have been synthesized; more precisely, the nature of their surface area, the conductivity of electrons, and long-standing stability, which contribute to higher conversion efficacy in the electrochemical reaction. GO has inherent electrocatalytic properties, and the implementation of this is mainly governed by the structure and configuration of the graphene sheets. The electrocatalytic performance of graphene-based electrocatalysts is reliant on the interfacial communication and intrinsic properties with the synergistic effect of homo/heterogeneous nanomaterials. In addition to this graphene based electrocatalysts acts as homogeneous polymer compounds as because of having functionalities to interact with other molecules in reaction media and also having its structural and morphological immovability which could make it possible to recycle and reuse. All these properties of GO makes an excellent electrocatalyst for higher conversion efficiency at low overpotentials.^23,24^

The main objective of this study was to investigate the electrochemical behavior of ETO by the determination and quantification of its oxidized form using PBS as the supporting electrolyte on modified GO electrodes. The electrochemical irreversible oxidation response is a novel platform to understand the oxidative conversion of ETO through an electron transfer mechanism. The oxidized form of ETO was confirmed by HRMS and FTIR and further evaluated for antibacterial activity against pathogens, such as *E. aerogenes*, *S. abony*, *S. boydii* and *E. coli*. The electrochemical synthesis and the improved biological activity of ETO have special importance in the pharma industry and studies of pharmaceutical products on a large-scale for other drugs.

## 2. Experimental methods

### 2.1 Reagents and solutions

Ethionamide (ETO) was purchased from Sigma Aldrich chemicals (99.9%). All other solvents and chemicals were of analytical grade and used without any further purification. The supporting electrolytes used in all the experiments were aqueous phosphate buffer at neutral pH (4 to 9.2), acetic buffer pH (3) and glycine NaOH buffer solution pH (11.2). The electrochemical studies were performed in phosphate buffer. Other reagents used were of analytical or chemical grade, and all solutions were prepared in Millipore water.

### 2.2 Synthesis of graphene oxide (GO)

Graphene oxide (GO) was synthesized from graphite powder using the modified Hummer's method. In a typical synthesis batch, 0.5 g of graphite powder was mixed with a mixture of acids containing 23 mL of nitric acid (97%) and 45 mL of sulfuric acid (98%) in a round-bottom flask and sonicated for 5 min, and the mixture was further stirred in an ice bath for 45 min. The dispersion was transferred into a round-bottom flask, which was then continuously sonicated for 3 h using an ultrasonicator (Citizon 50 Hz model). The sonicated solution was subjected to reflux for 20 h. Then, the reaction mixture was filtered and washed several times by continuously washing with distilled water until the pH of the filtrate became neutral. Moreover, it was again re-dispersed in THF to separate GO from unreacted graphite. Again, it was filtered and washed with acetone. The material was dried in an oven at 60 °C for 4 h.

### 2.3 Preparation of GO modified electrode

A glassy carbon (GC) electrode with a diameter of 3 mm was used as the working electrode. Prior to modification, the working electrode was cleaned with simultaneous polishing using 1.0, 0.3 and 0.05 μm size alumina powders, respectively, followed by washing with water and methanol to remove the inorganic and alumina impurities, respectively. Initially, the slurry was prepared with a calculated amount *i.e.* 0.07 mg of GO sample in 300 μL isopropanol and sonicated 3 h to make it homogeneous. Further, 2 μL (0.00046 mg) aliquots were drop-casted on GC and dried in the air, and it was used as the working electrode in further studies. The electrochemical studies were performed in aqueous electrolyte phosphate buffer at room temperature (25 °C ± 3 °C).

### 2.4 Instrumentation and analytical procedure

All electrochemical studies were performed on CHI-660E (CH-instruments USA) using a conventional three-electrode test cell with Pt foil, saturated calomel (SCE), and glassy carbon (GC) (3 mm dia.) as the counter, reference and working electrodes, respectively. Prior to the experiment, the working electrode was cleaned polishing using three different (grain sizes 1.0, 0.3 and 0.05 μm) alumina powders, followed by washing with water (deionized water) and methanol (AR grade) to remove the inorganic and organic impurities, respectively (the electrode was clean as confirmed by using it as a blank in cyclic voltammetry in the supporting electrolyte). All electroanalytical studies were carried out in aqueous phosphate buffer using three-electrode systems.

Linear sweep voltammetry (LSV) is a powerful electrochemical technique, which gives a signal of current at a specific potential, at which a species is oxidized or reduced. Further, electrochemical (oxidative LSV) studies were carried out by using 5 mM ETO in phosphate buffer as the supporting electrolyte within the potential range of −0.4 to 1 V *vs.* SCE. The scan rate dependency studies were performed using the LSV technique by sweeping from 2 to 300 mV s^−1^ using 5 mM ETO. The concentration dependency studies were carried out from 1 μmol L^−1^ to 100 mM concentrations of ETO at a scan rate of 10 mV s^−1^. The pH dependency studies were performed using different (pH 4 to 11.2) solutions in acidic, neutral and basic pH ranges using acetate, phosphate and glycine NaOH buffers respectively. The effect of excipients was tested in phosphate buffer at pH 7 with 2 mM ETO and excipients at 100-fold higher (100 mM) concentration.

*In vitro* antimicrobial activity of the synthesized compounds was evaluated by using the well-diffusion method reported by Phatak *et al.*^25^ In this study, potent bacterial and fungal pathogens were used. Both Gram-positive and Gram-negative pathogens were chosen for the studies. *Staphylococcus aureus* ATCC 6538, *B. cereus* ATCC 1177, and *Bacillus subtilis* ATCC 6633 were the Gram-positive pathogens used in this study. *Escherichia coli* ATCC8739, *Salmonella typhi* ATCC9207, *Shigella boydii* ATCC 12034, *Enterobacter aerogenes* ATCC13048, *Pseudomonas aeruginosa* ATCC9027, and *Salmonella abony* NCTC6017 were the Gram-negative pathogens, and *Aspergillus niger* ATCC 16404, *Saccharomyces cerevisiae* ATCC 9763, and *Candida albicans* ATCC10231 were the fungal pathogens. Fluconazole and streptomycin were used as the antifungal and antibacterial reference compounds, respectively. All compounds were dissolved in DMSO at a concentration of 1 mg mL^−1^. For preparing fresh inocula of the bacterial and fungal pathogens, each bacterium and fungi was inoculated in sterile Mueller Hinton broth and incubated at 37 °C for 24 hours for growth, and this broth with well-grown pathogens was used for further studies. Using sterile saline, the bacterial suspension was diluted to adjust the turbidity to the 0.5 McFarland standards. 200 μL diluted suspension of each pathogen was inoculated on sterile Mueller Hinton agar plates. Wells were punched in the agar medium. Using a micropipette, 100 μL of each compound solution was placed in separate wells. 100 μL of DMSO solution without any compound was also placed in a well to check its activity against the pathogenic culture. All Petri dishes were incubated for 24 h at 37 °C. A clear zone around the well was considered a positive result. After complete incubation, the antimicrobial activity of the synthesized compounds was measured. Zones were measured and recorded in millimeters (mm) by using a scale.

### 2.5 Characterization

X-ray diffraction (XRD) was carried out on a Rigaku Ultima IV fully automatic high-resolution X-ray diffractometer system with the X-ray generator operating at 40 kV and 40 mA at steps of 0.01° (2*θ*) at room temperature. Raman spectroscopy was performed by Raman optics using a microscope from Seki Technetronic Corporation, Tokyo with a 532 nm laser. Scanning electron microscopic (SEM) measurements were carried out with a JEOL (JSM-7600F) instrument equipped with an energy dispersive X-ray (EDX) attachment. The antifungal activity of the synthesized compounds was determined against the fungal pathogens *Aspergillus niger* ATCC 16404, *Saccharomyces cerevisiae* ATCC 9763, and *Candida albicans* ATCC10231. Fluconazole and streptomycin were used as the antifungal and antibacterial reference compounds, respectively. All compounds were dissolved in DMSO at a concentration of 1 mg mL^−1^. Each bacterium and fungi was inoculated into sterile Mueller Hinton medium and incubated at 37 °C for 24 h for developing the inocula, and thereafter, these broths were used in the studies. Using sterile saline, the bacterial suspension was diluted to adjust the turbidity to the 0.5 McFarland standards. 200 μL diluted suspension of each pathogen was inoculated on sterile Mueller Hinton agar plates. Wells were punched in the agar medium. Using a micropipette, 100 μL of ETO solution was placed in a separate well. 100 μL of DMSO solution without any compound was also separately placed in a well to check its activity against the pathogenic cultures. All Petri dishes were incubated for 24 h at 37 °C. A clear zone around the well was considered a positive result. After complete incubation, the antimicrobial activity of the synthesized compounds was measured. Zones were measured and recorded in millimetres by using the scale (mm).

## 3. Results and discussion

### 3.1 Structural and morphological analysis

**Fig. 1:**
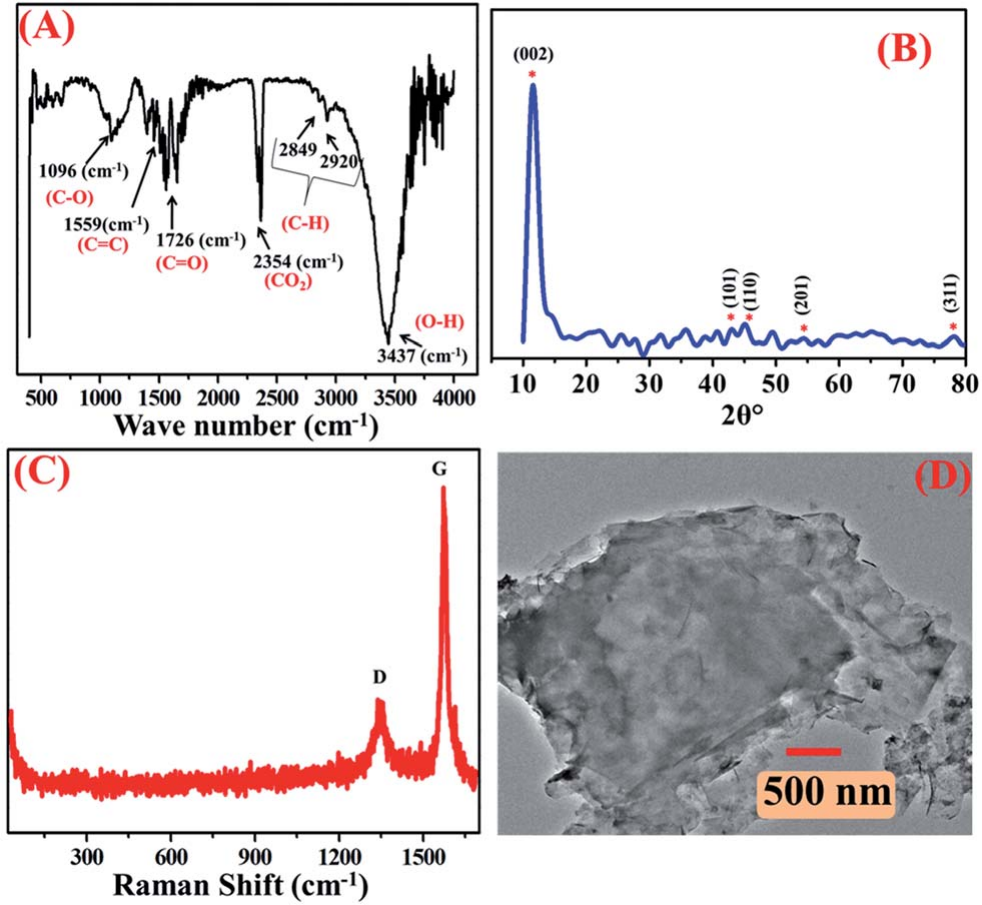
(A) FTIR spectra of GO, (B) XRD pattern of GO, (C) Raman spectrum and (D) TEM image of GO.

The as-synthesized GO was well-characterized by structural and morphological techniques, namely FTIR, Raman spectroscopy, XRD and TEM, respectively, as shown in Fig. 1. Accordingly, the FTIR spectrum was used as a tool to confirm the oxidative exfoliation of graphite powder to GO and as shown in Fig. 1(A). The representative broad and sharp peaks demonstrated different oxygen functionalities generated on GO. The peaks observed at 3437, 2920, 2849, 2354, 1726, 1559 and 1096 cm^−1^ corresponded to O–H, C–H and the carboxylic functional groups *i.e.* carbon skeleton and oxidative functionalities, namely CO_2_, C=O, C=C and C–C, respectively. All these characteristic functional groups of GO matched well with the literature, confirming that GO was functionalized successfully.^26^ XRD analysis is a powerful technique to determine the crystalline nature of materials. It also helps to determine the spacing between the layers, as well as the orientations of the materials. Accordingly, the formation of GO was confirmed from the signals that appeared in the XRD pattern, as shown in Fig. 1(B). The diffraction angle 2*θ* values observed at 10.09°, 42.13°, 44.16°, 54.13° and 77.29° corresponded to the (002), (101), (110), (201) and (311) planes, respectively, confirming GO formation and are also in good agreement with the FTIR results (Fig. 1(A)) and literature.^27^ The Raman spectrum showed specific information concerning GO as the G-band resulted from the sp^2^ carbon network and the D-band corresponded to the defected E_2g_ carbon. Fig. 1(C) demonstrates the characteristic Raman spectra of GO with the D-band related to the disorder in graphene at ∼1327 cm^−1^ and the G-band corresponding to the C–C bond stretching in GO at ∼1560 cm^−1^, and their representative intensity ratio (*I*_D_/*I*_G_) was observed to be 0.28. From the Raman spectrum, it was clear that graphite with defected graphene oxidized to GO.^28^

The morphological was determined by transmission electron microscopy (TEM). The TEM images, as shown in Fig. 1(D), revealed a wrinkled and folded structure of graphene with a few layers with thickness of 500 nm. The difference between the two layers was observed to be 50 (±5) nm due to the defected sp^3^-hybridized carbon with a different functional group, which is quite similar to other reports.^29^

**Fig. 2:**
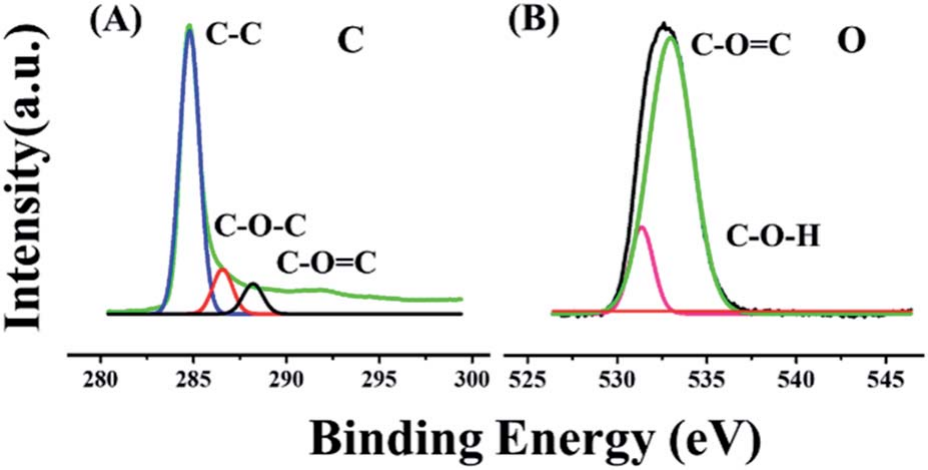
Superimposed high resolution XPS spectra of the as-synthesized GO (A) C 1s spectra related to the three kinds of carbon (C–C, C–O–C and C=O), (B) O 1s ascribed to (O–H and C–O) bonding interactions.

Additionally, X-ray photoelectron spectroscopy (XPS) was employed to recognize the surface alignment and the chemical state of the elements present on the surface. From Fig. 2(A), it can be observed that the graphene sheets contained C and O elements. In addition to this, three types of carbon existed. The intense peak at 284.2 eV was attributed to asymmetric C–C, the peak at 286.00 eV was associated with the C–O–C bonding and that at 288.2 eV was related to the C=O bonding in all the oxygen encompassing functional groups existing on the graphene sheet. Further, the O 1s core with a signal at 530.1 eV revealed the presence of C–O–H, and the signal at 533.4 eV corresponded to C–O,^30,31^ which could be present in GO, as shown in Fig. 2(B). The electron rich graphene oxide enhances the mobility of electrons and conversion efficiency. Due to the presence of oxygen-containing species, it alters the surface properties of graphene and results in a lower resistance frequency, as discussed in Section 3.2 to support these observations.

### 3.2 Electrochemical oxidation of ETO

**Fig. 3:**
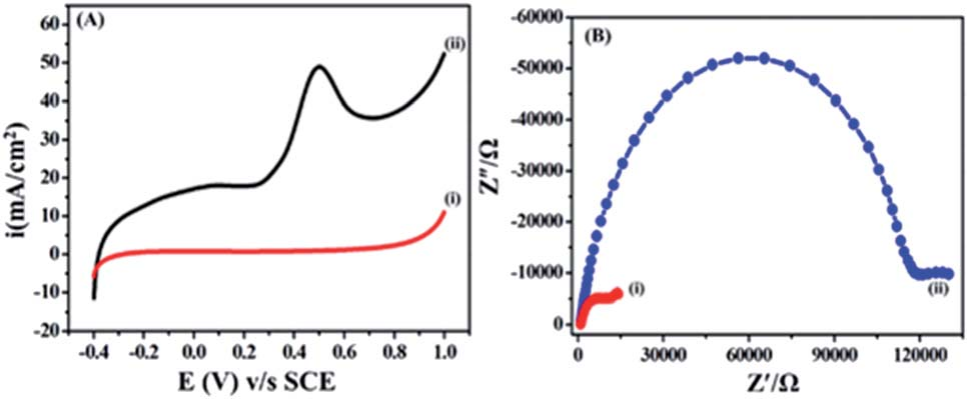
(A) Electrochemical behaviour of 5 mM ETO on (i) bare GC and (ii) GO/GC working electrode at a scan rate of 10 mV s^−1^ in PBS buffer. (B) Electrochemical impedance data of (i) GO modified GC and (ii) bare GC in 0.1 M buffer at pH 9.2 containing 5 mM ETO in 0.01 to 500 Hz frequency range.

The electrochemical behaviour of ETO was investigated on GO (modified GC working electrode) electrode by the linear sweep voltammetric (LSV) technique at a sweep rate of 10 mV s^−1^ in PBS buffer (9.2 pH) over the potential range of −0.4 to 1 V, as shown in Fig. 3(A). Accordingly, the anodic sweep of 5 mM ETO on the GO electrode gave a characteristic irreversible anodic signal at 0.240 V, corresponding to the oxidation of ETO, which was confirmed by the featureless LSV for blank measurements *i.e.* without the addition of ETO. Moreover, the significant increase of anodic current density on GO suggested that electron transfer in GO is more feasible than that in bare GC for the oxidation of ETO. Furthermore, we compared the anodic LSV curves from bare GC electrode, bare GO/GC electrode, GC electrode with 5mM ETO and GO/GC electrode with 5 mM ETO in pH 9 phosphate buffer electrolyte at 10 mV s^−1^, as shown in Fig. S4 in ESI.†

The electrocatalytic activity of graphene was independent of the number of graphene sheets as there was no significant change between the electrochemical performances of single- and multiple-layer graphene samples because the number of edge planes remained unchanged. However, there was a significant difference in activity between the solid carbon electrode and the graphene modified electrode because graphene has a higher number of edge planes per unit mass *i.e.* surface area. As a result, the electrocatalytic performance was altered by an increase in current density, with decreasing overpotentials.^32^

The literature significantly reveals that GO has enormous potential in applications, as it has shown enhanced electrocatalytic activity than those of pristine graphene and the bare electrode. Ming-Chin Yu group reported the electrochemical detection of the anti-cancer drug flutamide on a GO modified glassy carbon electrode. The study also demonstrated faster electron transfer in the GO modified electrode compared to the bare GC electrode by showing a wider linear response range, an excellent limit of detection and good sensitivity.^33^ Matsumoto *et al.* studied GO-based paper proton membrane fuel cells at relative temperatures, and the results demonstrated that, due to the incorporation of GO paper in the cell, the current density increased.^34^ Moreover, Li *et al.* summarized a review on the electrocatalytic synthesis of GO by different methods and the properties and applications of GO. The review critically deliberates the current advances in the use of GO-based nanomaterials over pristine graphene and graphite electrodes in the fields of electroanalytical chemistry and electrochemical sensors.^35^

Electrochemical impedance is commonly used to decide the interface resistance and feasibility of electron transfer at the electrified interfaces of GO. The electrochemical impedance of GO modified GC and bare unmodified GC was measured in a buffer at pH 9.2 containing 5 mM ETO from 0.01 to 500 Hz, as shown in Fig. 3(B). A semi-circle was observed due to charge transfer resistance (*R*_ct_) at the electrolyte interface and electrode. The EIS plot showed a lower charge transfer resistance (14 022 Ω) on GO compared to GCE (130 029 Ω). The Nyquist plots were also supportive of our earlier findings from the LSV method and the diffusion-controlled electron transfer in GO.^36^ In addition to this, we performed an experiment with 100 cycles of cyclic voltammetry on the GO modified GC electrode in phosphate buffer at pH 7 and a scan rate of 50 mV s^−1^ to assess the stability of the functionalized GO and different oxygen groups present on GC. The results in Fig. S5 in ESI† show that there was no significant change in CV after 100 cycles. The results also demonstrated that the oxygen content in GO did not change during cycling.

### 3.3 Effect of accumulation time on current

**Fig. 4:**
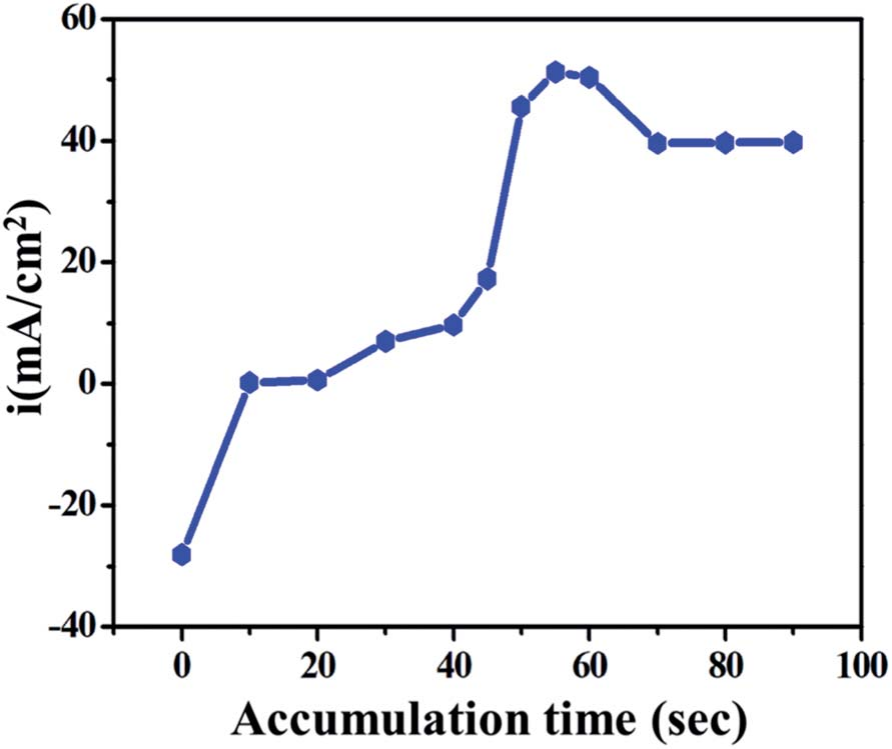
Effect of accumulation time on current density.

To improve the electroanalytical limit of detection, sensitivity and time taken to oxidize the drug on the electrode, the effect of accumulation was studied. The effect of accumulation time on the anodic current was studied using the LSV technique on GO/GC in phosphate buffer under 10 mM ETO concentration. The plot of current *vs.* accumulation time was generated for 0 to 90 seconds, as shown in Fig. 4. It was concluded that the number of drug molecules adsorbed on the electrode and accordingly, the current increase with time until about 50 s, whereas the current decreases with a further increase in accumulation time and reaches a limiting value.

Initially, until about 0 to 10 seconds, the current slightly increased due to non-faradic current or only due to the mass transfer process; it was constant for the next 40 seconds. At ∼55 seconds, the current reached the maximum value because of the faradic electron transfer process *i.e.*, the anodic oxidation of the drug molecule on the electrode. Following this, the current was stable until 90 seconds as it reached the limiting value. 55 seconds was considered as the working time in further studies.

### 3.4 Effect of pH

**Fig. 5:**
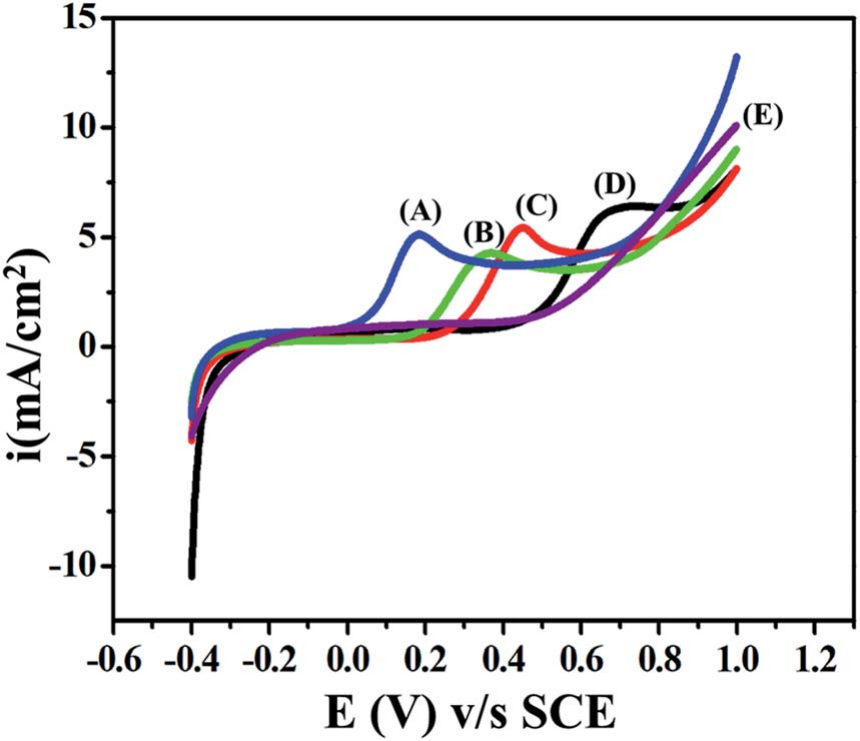
Influence of pH on the shape of the anodic peak at pH: (A) 11.2, (B) 9.0, (C) 7.0, (D) 5.0 and (E) 3.2.

pH is an important parameter for biologically active analytes. It is directly associated with the electron and proton transfers, which can promote and demote electrochemical reactions. Here, the pH reliance of 5 mM ETO was studied with different buffers: acetate for acidic (4 pH), PBS for neutral (5–9) and glycine NaOH (9–11.2) for basic pH ranges. At lower pH values, the anodic peak potential of ETO shifted towards higher potential values because of the instability of the reactant molecules.^37^ However, at higher pH values, the onset potential shifted towards lower values as the OH^−^ ion concentration was high, which favours the anodic reaction at the electrode surface in the basic pH range, as shown in Fig. 5. The sulphide reaction centre is conjugated with the pyridinic nitrogen lone pair of electrons which stabilizes the electron density, forming an electrophile. Secondly, sulphur is easily oxidized at lower potentials because of the electronic hindrance due to the dense 3p orbitals at high pH, and due to the high OH^−^ concentration, the potential shifts towards negative values. Furthermore, at low pH, the pyridinic nitrogen gets hydrogenated, which destabilizes the intermediate, and a positive shift is observed. The peak potential and pH (3.2 to 11.2) show a linear relationship, as the peak potential value shifts positive or negative depending on the pH, while the anodic peak current remains almost constant. The secondary graph of potential *vs.* effect of pH clearly indicates that pH strongly affects the onset potential of ETO oxidation.

### 3.5 Effect of scan rate

**Fig. 6:**
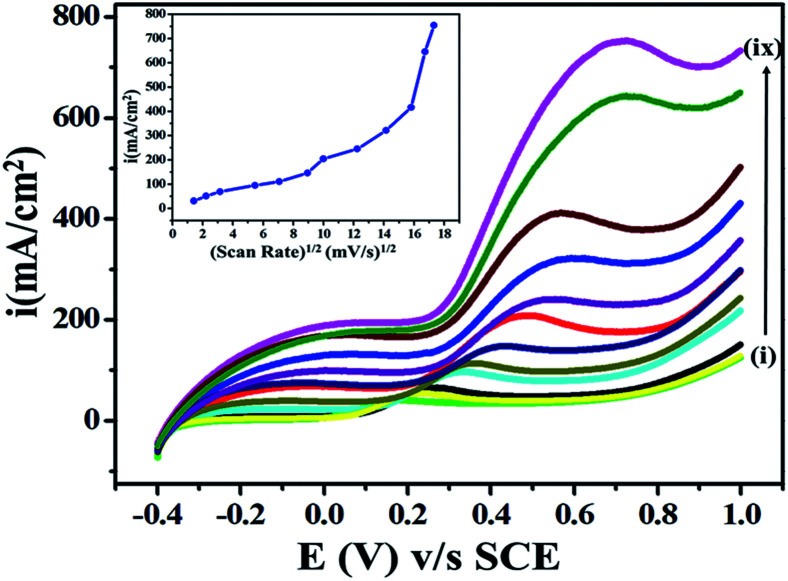
Superimposed LSV curves showing the effect of scan rate (i–ix): 2, 5, 10, 50, 100, 150, 200, 250 and 300 mV s^−1^ on the anodic peak current for ETO (5 mM) oxidation in pH 9.2 buffer as the supporting electrolyte.

The effects of scan rate on current and potential are among the important parameters in an electroanalytical experiment, as they influence the diffusion-controlled electron processes during the electrooxidation of ETO. Accordingly, the effect of the potential sweep rate on (5 mM) ETO oxidation on GO was studied in the scan rate range 2 mV s^−1^ to 300 mV s^−1^. The current significantly increased as the potential sweep rate increased, as shown in Fig. 6, which suggested that the oxidation of ETO on GO is diffusion-controlled. As the scan rate increased, the anodic segment potential value shifted gradually towards higher positive values, probably due to the high charge transfer rate in the electroactive drug molecules, which is restricted to the electrode surface only.^38^

### 3.6 Analytical application calibration curve (concentration dependent)

**Fig. 7:**
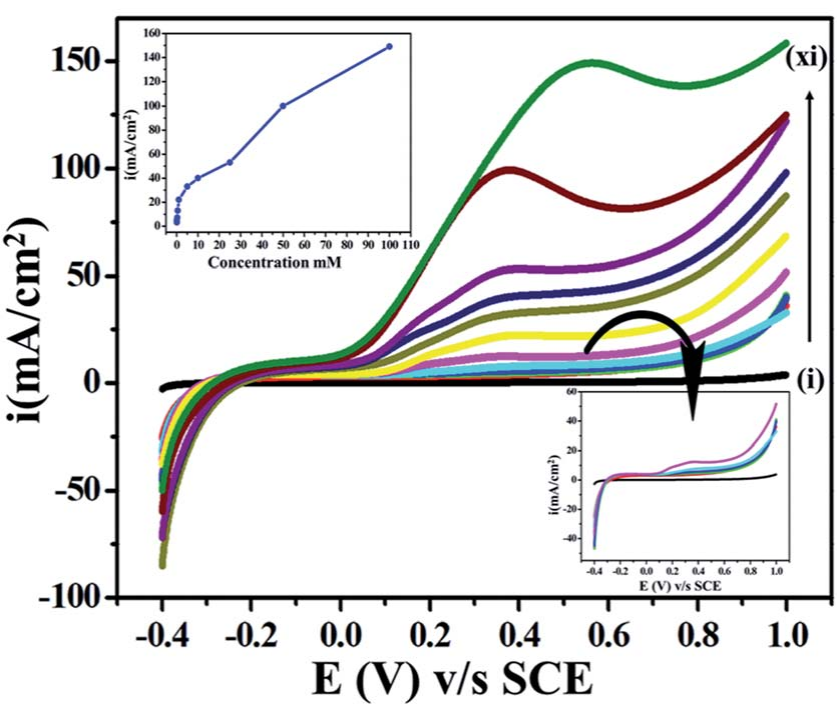
Superimposed LSV curves of the GO electrode with various concentrations of ETO (i–xi): bare GC, 0.001, 0.01, 0.1, 0.5, 1, 5, 10, 25, 50 and 100 mM in phosphate buffer as the supporting electrolyte at 10 mV s^−1^.

For the evaluation of the feasibility of electron transfer and analytical application, we performed concentration dependency studies, as shown in Fig. 7. We found an increase in the current response directly proportional to the concentration of ETO (1 μM to 100 mM) in PBRS (pH 9.2) at the scan rate of 10 mV s^−1^. Excessive current density was observed at high concentrations due to the availability of a higher number of faradic electro-active species on the electrode surface for electron transfer. Moreover, the onset potential shifted towards negative values as the concentration of the drug increased, and the reason for this is the easy accessibility of ETO molecules on the electrode surface.

Furthermore, in order to provide a quantitative determination of the drug, LSV with a low scan rate *i.e.*, 10 mV s^−1^ was employed to enhance the selectivity for ETO. A linear concentration succession was observed in the 1 μM to 100 mM concentration range. Additionally, the limit of detection (LOD) and limit of quantification (LOQ) were determined. LOD is the lowest analyte concentration, at which detection is reasonable. LOQ is the lowest concentration, at which the analyte can be constantly detected with a linear response. LOD and LOQ were calculated by using the following equations:LOD = 3*s*/*m* and LOQ = 10*s*/*m*where, ‘*s*’ is the standard deviation of the intercept, and *m* is the average slope of the regression line.

For this GO-based system, the LOD was found to be 1.33, and the LOQ was also calculated as 4.4. The lower values of LOD and LOQ demonstrate that the system was significantly sensitive for ETO detection on the metal-free electrocatalyst in an aqueous medium. The LOD of this electrochemical method is lower compared to other methods reported in the literature, which confirms that this system is more precise and sensible, as shown in Table S1 (ESI, ref. 1–4†). In addition, this GO electrochemical sensor was compared with various electrochemical methods reported in the literature for ETO determination, as shown in Table S2 (ESI, ref. 5–7†).

### 3.7 Effect of excipients

The effect of excipients was studied using different biologically active molecules at a 100-fold (100 mM) higher concentration than that of ETO (2 mM). Table 1 shows that there was no significant change in the onset potential of ETO oxidation. A major change was observed only with citric acid, and the reason may be that, with the addition of citric acid, the pH of the solution changed acidic, which directly had an effect on the onset potential of ETO oxidation. The results demonstrate that the method was greatly selective for the determination of ETO since even when common biological excipients were added, there was no current change in the anodic peak.

**Table 1:** Influence of potential on (2 mM) ETO with different excipients (100 mM)

Excipients (100 mM) + drug ETO (2 mM)	Potential (V)	Signal change (%)
Only ETO	0.059	0
Sucrose + ETO	0.056	5.09
Starch + ETO	0.055	6.47
Glucose + ETO	0.064	8
Citric acid + ETO	0.0343	42
Ascorbic acid + ETO	0.055	6.47
Dextrose + ETO	0.053	11.27

### 3.8 Complete oxidation of ETO (conversion to ETO-O)

**Fig. 8:**
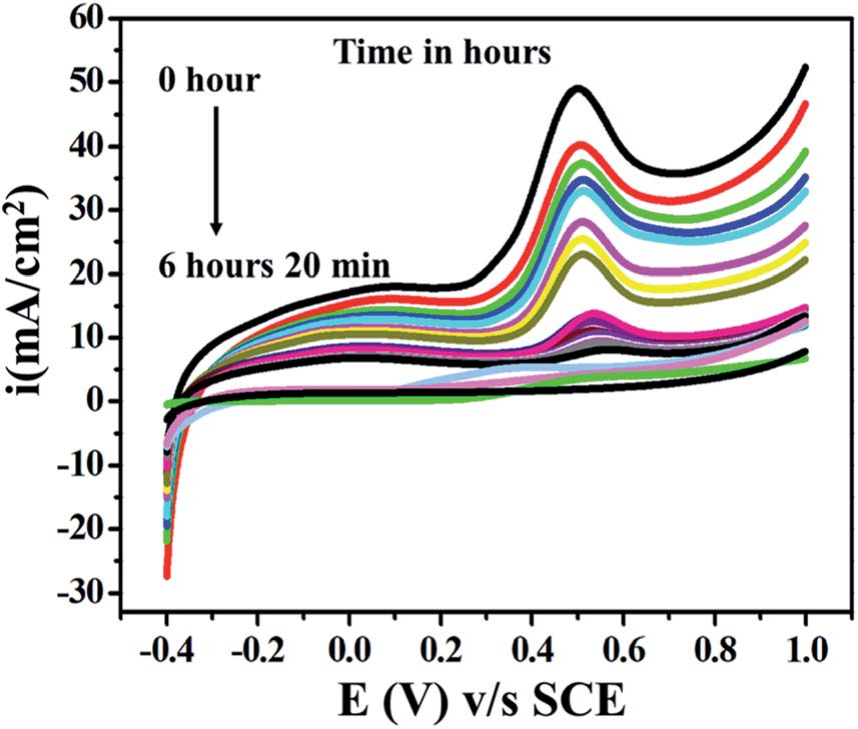
Superimposed LSV curves recorded for the complete oxidation of (5 mM) ETO on GO electrode *vs.* SCE in 100 mL of pH 9.2 buffer at the scan rate of 10 mV s^−1^.

For characterization and biological evaluation, we carried out complete oxidation (conversion) of ETO to its oxidized form 4-amino-5-hydroxymethyl-2-methylpyrimidine under similar experimental conditions. Continuous LSV measurements of ETO (5 mM) were recorded on GO at a sweep rate of 10 mV s^−1^ in PBS buffer (9.2 pH) over the potential range −0.4 to 1 V. As complete oxidation occurred at about 6.2 h, the anodic peak decreased significantly and reached zero, as shown (a few representatives) in Fig. 8.

The significant decrease in anodic peak indicated the complete oxidation of ETO to its oxidized form. Furthermore, the product was recrystallized using ethanol to carry out further characterization studies and biological activity evaluation. In our previous report, we have demonstrated the determination and oxidation of emtricitabine. The study also demonstrated that the biological activity of the oxidized product increased significantly.^39^ Additionally, the crystallized compound was characterized by HRMS spectroscopy for confirmation and to identify the exact bonding present in it. In addition to this, the HRMS spectrum further strengthened the structure assigned to 4-amino-5-hydroxymethyl-2-methylpyrimidine (2), showing an [M]^+^ ion corresponding to *m*/*z* 152.0711 for the molecular formula C_8_H_12_N_2_O, as shown in ESI Fig. S1.†

**Fig. 9:**
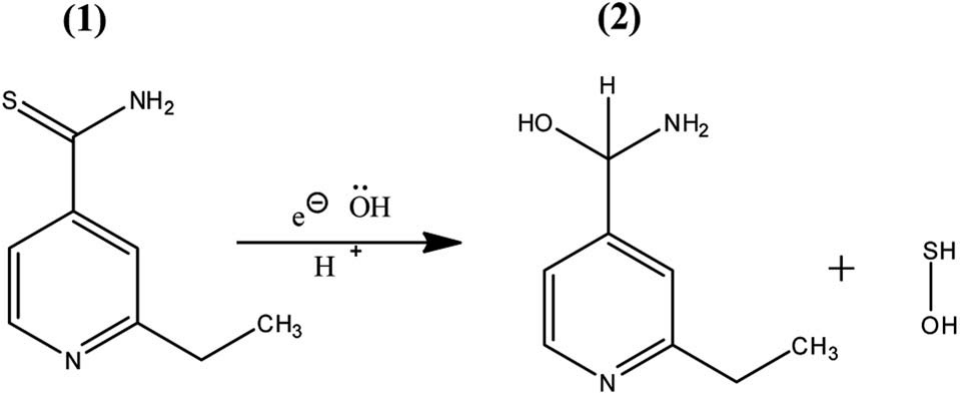
Structures of (1) the pristine drug ETO and the (2) ETO-oxidised form, 4-amino-5-hydroxymethyl-2-methylpyrimidine.

Furthermore, we carried out FTIR spectroscopic characterization of the oxidized compound and ETO in the 500 to 4000 cm^−1^ range, as shown in ESI Fig. S2.† The stretching frequencies observed were between 3175 and 3182 cm^−1^ for N–H, 1287 cm^−1^ and 825 cm^−1^ for C=S stretch, and some additional frequencies for the conjugation and aromatic systems, which were in good agreement with the literature.^40^ The oxidised form contained an additional O–H stretch at 3375 cm^−1^ whereas C=S stretching was not found, clearly indicating the loss of sulphur group with nucleophilic OH^−^.^41^ The FTIR results are in good agreement with the above-displayed HRMS spectra, which confirmed the oxidation of ETO to ETO-O (Fig. 9).

### 3.9 Mechanism

The electrooxidation of ETO to on the GC/GO modified electrode in buffer is an irreversible oxidation process. On the basis of the electrochemical studies and product confirmation by characterization techniques, we confirmed the formation of 4-amino-5-hydroxymethyl-2-methylpyrimidine. The plausible mechanism for the conversion of ETO to 4-amino-5-hydroxymethyl-2-methylpyrimidine is given in ESI Fig. S2.† Generally, sulfenic acids and sulphur oxides have less stability in the absence of bulky groups in the surrounding sulphur centre. Few organosulphur compounds are stable in the S-hydroxide form when the sulphur has a zero oxidation state center, *e.g.* ethionamide, which can be isolated.^42^ Here, the sulphide carbon acts as a nucleophilic centre conjugated with the pyridinic nitrogen lone pair of electrons, which stabilize the electron density from the electrophile. Secondly, sulphur is easily oxidized at lower potentials because of the electronic hindrance due to its dense 3p orbitals.^43,44^ Overall, nucleophilic displacement reaction with the –OH group, followed by the breaking of the C–S bond and the formation of 4-amino-5-hydroxymethyl-2-methylpyrimidine with an S–OH by-product happens at the anodic electrified interface. Based on the FTIR, HRMS and electrochemical results, we have narrated the plausible detailed mechanism, as shown in ESI Fig. S2.† Overall, the hydroxyl (–OH) lone pair of electrons (nucleophile) radially attacks the sulphide carbon, which acts as an electrophilic carbon; with the subsequent addition of the –OH nucleophile and breaking of the C–S bond, elimination of SH and the formation of 4-amino-5-hydroxymethyl-2-methylpyrimidine product and SH-OH side product occur.^45–47^

### 3.10 Antimicrobial activity

ETO is a standard anti-tubercular drug, that doesn't exhibit antimicrobial activity against any bacterial and fungal pathogens. On the contrary, the modified form (oxidized) ETO-O (4-amino-5-hydroxymethyl-2-methylpyrimidine) of the drug did exhibit antimicrobial activity, but only against some pathogens. ETO-O showed good activity against some Gram-negative pathogens, such as *E. aerogenes*, *S. abony*, *S. boydii*, and *E. coli* but was not effective against Gram-positive bacterial pathogens and fungal pathogens,^25^ as shown in Table 2. The photographs of the antibacterial activity assay are shown in Fig. S6 in ESI.†

**Table 2:** Results of antimicrobial assay of the synthesized compounds against potent pathogens. Diameter of the zone of inhibition is given in millimeter (mm). The standards used for antibacterial and antifungal activity were streptomycin and fluconazole, respectively

Pathogens	Compounds		
	ETO	ETO-O	Standard
*S. typhi* ATCC9207	00	10	33
*E. aerogenes* ATCC13048	00	05	29
*B. subtilis* ATCC 6633	00	06	32
*C. albicans* ATCC10231	00	00	30
*P. aeruginosa* ATCC9027	00	06	32
*S. abony* NCTC6017	00	12	32
*E. coli* ATCC8739	00	10	29
*S. aureus* ATCC 6538	00	00	29
*S. boydii* ATCC 12034		11	34
*S. cerevisiae* ATCC 9763			30
*A. niger* ATCC 16404			30
*B. cereus* ATCC 1177		06	33

The compound ETO-O was effective against Gram-negative bacterial pathogens only but not against Gram-positive bacterial pathogens and fungal pathogens. This might be due to the differences in cell wall composition between the Gram-positive, Gram-negative and fungal pathogens. Being a prokaryotic cell, Gram-positive bacteria possess a high content of peptidoglycan than Gram-negative bacteria. As fungal cells are eukaryotic in nature, their cell walls are totally different from bacteria. Most antifungal agents target ergosterol, an important component in the fungal cell wall. Antimicrobial agents inhibit or kill pathogens by inhibiting DNA synthesis, protein synthesis or cell wall synthesis. After scrutinizing the result, it is evident that ETO-O probably acts by attacking the cell wall.

Surface active agents produce an alteration in zeta potential that can be correlated with permeability differences. These compounds increase their electrostatic affinity towards the cell surface and thereby, increase the surface permeability.^48,49^ Gram-positive and Gram-negative bacteria differ in their responses to such compounds/alteration in zeta potential. Gram-positive bacteria possess more negative charges due to altered zeta potential and also show more hydrophobicity, whereas Gram-negative bacteria do not show increased hydrophobicity. This difference can be attributed to the cell wall structure of these organisms. Gram-positive bacteria possess a thick cell wall along with a cover of the outer membrane. The presence of a large amount of peptidoglycan makes their cell wall thicker. However, in Gram-negative cell walls, the amount of peptidoglycan is less and negatively charged lipopolysaccharide is present. Interaction between surface active agents and the negatively charged polysaccharide –O^−^ side chains results in altered permeability of the Gram-negative cell membrane. The cation-selective barrel protein also mediates the internalization of cationic compounds and thereby, increases the permeability and eventually results in cell death. Gram-negative bacteria are more susceptible than Gram-positive.^50^

## 4. Conclusion

Determination of the electrochemical oxidation of ETO was successfully carried out on a GO-based metal-free electrode system by using aqueous phosphate buffer as the supporting electrolyte. The synthesized GO electrode was well characterized by FTIR, XRD, Raman spectroscopy and TEM. LSV and impedance techniques were employed in this study under optimized conditions. Significantly, the GO-based anode showed a linear range and diffusion-controlled electron transfer path with an LOD of 1.33 and LOQ of 4.4, for the analytical determination of ETO. Furthermore, complete electrochemical oxidation of ETO was achieved within 6 h 20 min, and the product was confirmed by high resolution mass spectroscopy (HRMS) and Fourier transmission spectroscopy (FTIR). 4-Amino-5-hydroxymethyl-2-methylpyrimidine (ETO-O) was evaluated for anti-bacterial activity, and the results displayed good activity against some Gram-negative pathogens, such as *E. aerogenes*, *S. abony*, *S. boydii*, *E. coli*, while it was not found effective against Gram-positive bacterial pathogens and fungal pathogens. This metal-free environmental friendly catalyst approach is applicable for the determination and biological evaluation of other drug molecules also.

## Conflicts of interest

There are no conflicts to declare.

## Supplementary Material

RA-009-C9RA06681K-s001
