# Supplementary material for: Graphene oxide-based electrochemical activation of ethionamide towards enhanced biological activity
Source: RSC Adv. 2019 Nov 1;9(61):35463–72. doi: 10.1039/c9ra06681k (PMC9074427; doi:10.1039/c9ra06681k)
Supplement: RA-009-C9RA06681K-s001 [file RA-009-C9RA06681K-s001.pdf]

Electronic Supplementary Material (ESI) for RSC Advances.
This journal is © The Royal Society of Chemistry 2019

Supporting Information

Graphene Oxide based Electrochemical Activation of Ethionamide towards Enhanced Biological Activity

Balaji B. Mulik[a], Sambhaji T. Dhumal[a], Vijay S. Sapner[a], Naziya N.M.A Rehman[b], Prashant P Dixit[b] and Bhaskar R. Sathe[a]

[a]*Department of Chemistry, Dr. Babasaheb Ambedkar Marathwada University, Aurangabad 431004, Maharashtra, India*

[b]*Department of Microbiology Dr. Babasaheb Ambedkar Marathwada University Aurangabad, Sub-campus, osmanabad.413501.MH.India.*

Email: bhaskarsathe@gmail.com

Fig. S1. HRMS spectrum of ETO-O (4-Amino-5-hydroxymethyl-2-methylpyrimidine) showing $[M]^+$ ion peak at m/z 152.0711 for its molecular formula $C_8H_{12}N_2O$.

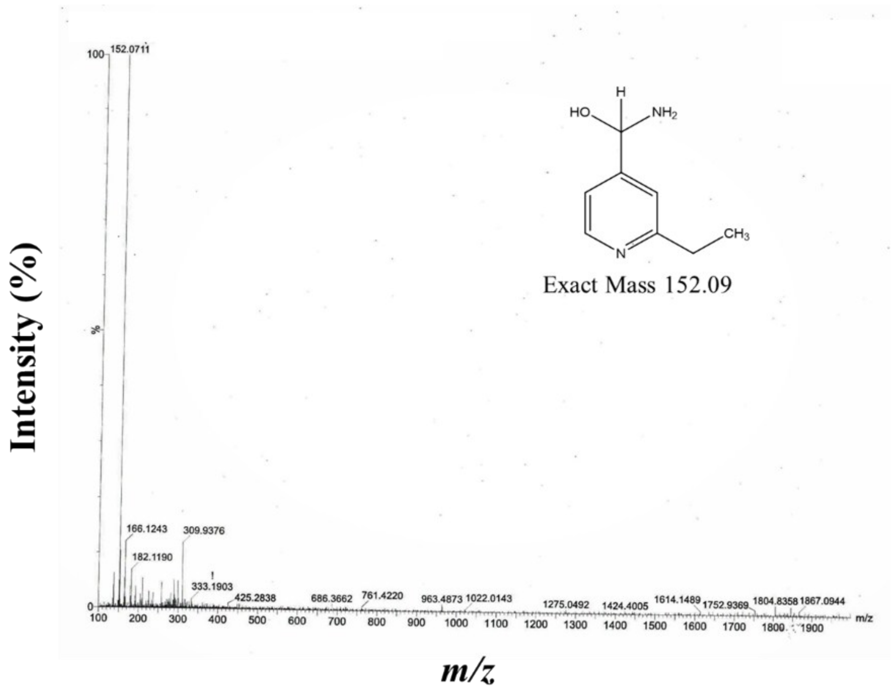

Fig. S2. FTIR spectrum of ETO and oxidized form of ETO (4-Amino-5-hydroxymethyl-2-methylpyrimidine) by using KBr powder (IR grade).in the range 500 to 4000 cm^{-1} at scan rate 30 mV/s, showing the characteristics band for conformation of oxidation of ETO.

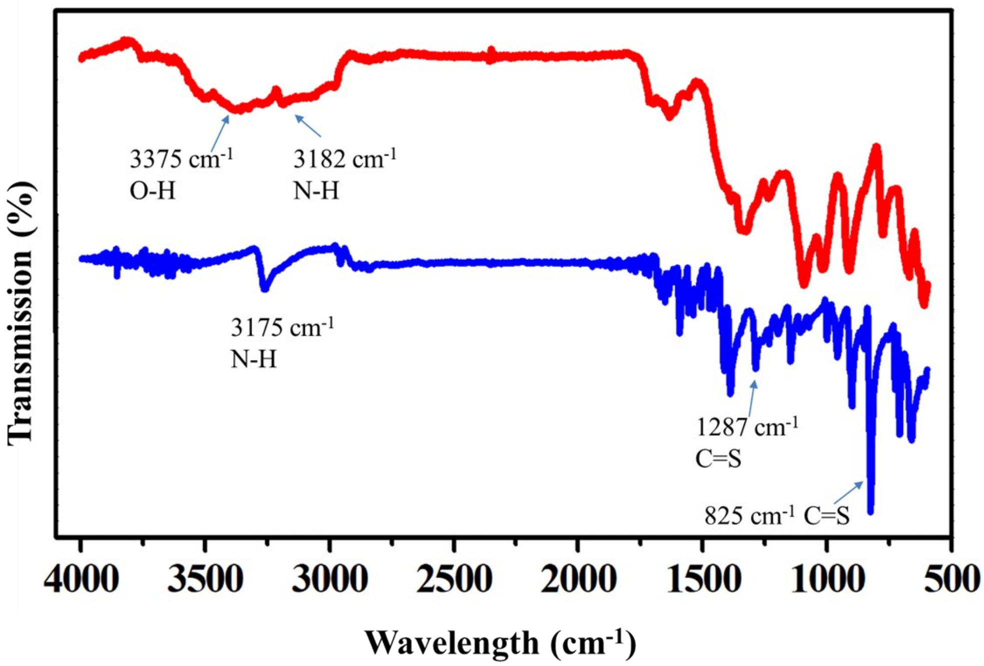

Fig. S3. Plausible detailed mechanism for ETO to 4-Amino-5-hydroxymethyl-2-methylpyrimidine

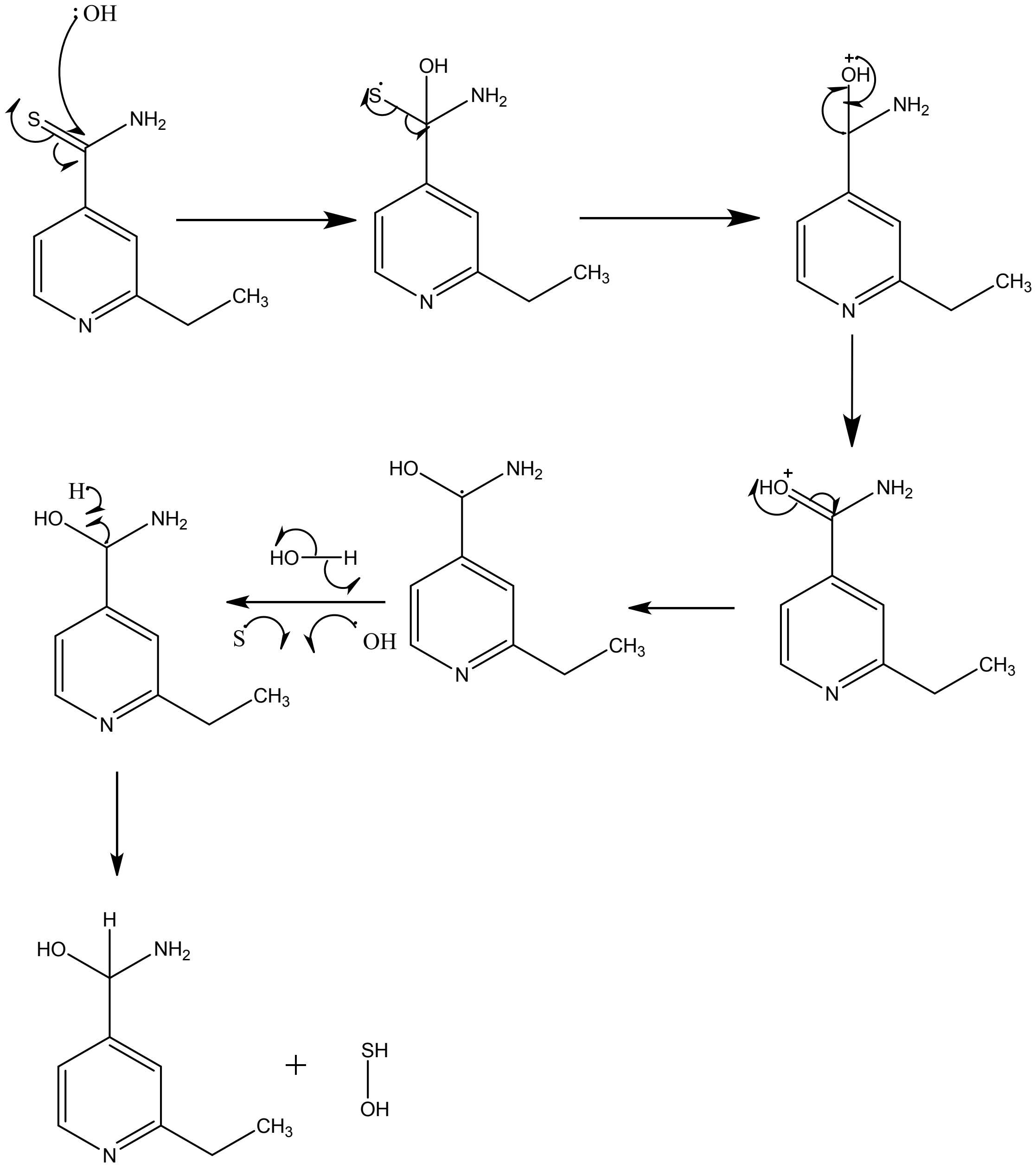

Fig. S4. Superimposed LSV anodic Curves (i) bare GC electrode, (ii) Bare GO modified GC electrode, (iii) bare GC electrode with 5 mM ETO, (iv)GO modified GC electrode with 5 mM ETO in pH 9 (1M) phosphate buffer electrolyte at 10 mV/s scan rate.

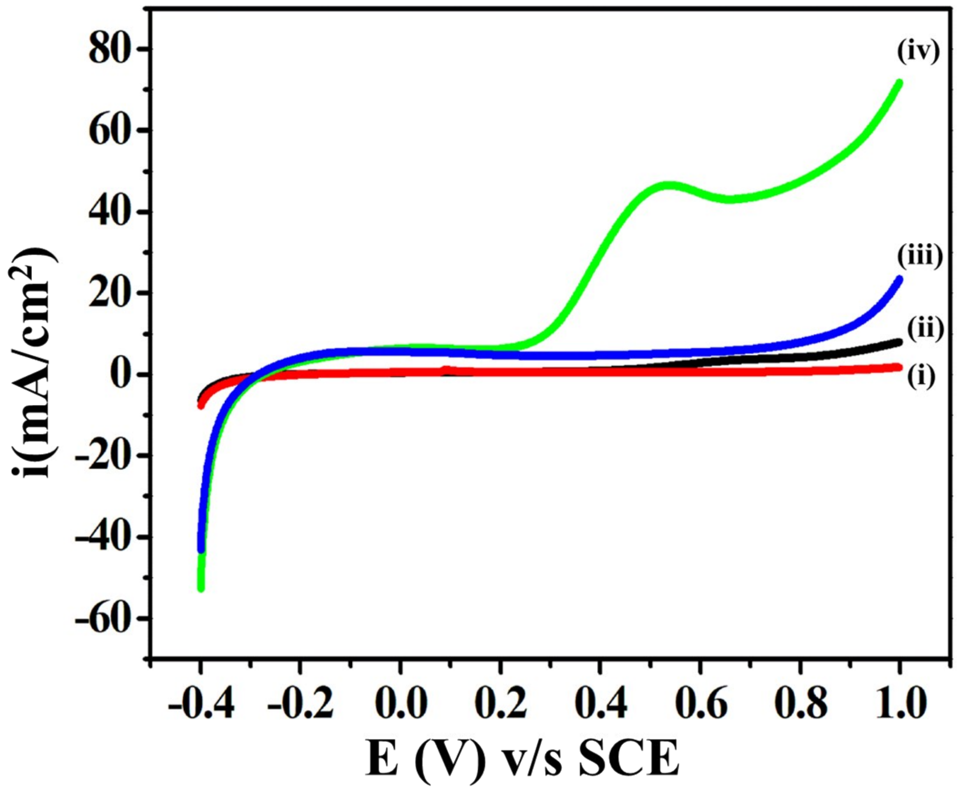

Fig. S5. Superimposed Cyclic Voltametric (CV) Curves GO modified GC electrode, in pH 7 (1M) phosphate buffer electrolyte at 50 mV/s scan rate.

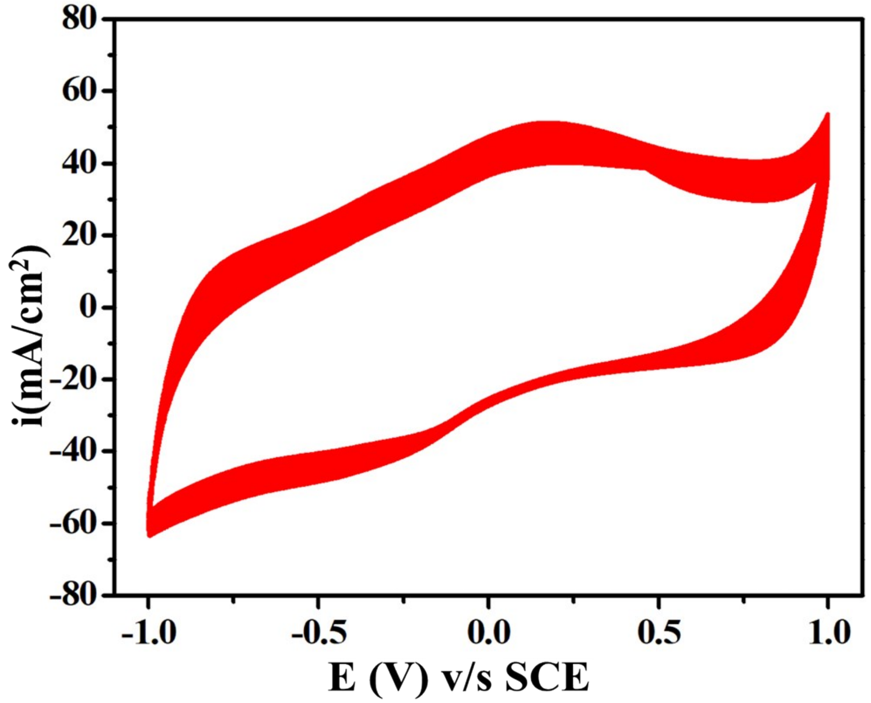

Fig. S6. Antimicrobial activity of ETO1, ET. Streptomycin is used as standard (positive) compound for this assay.

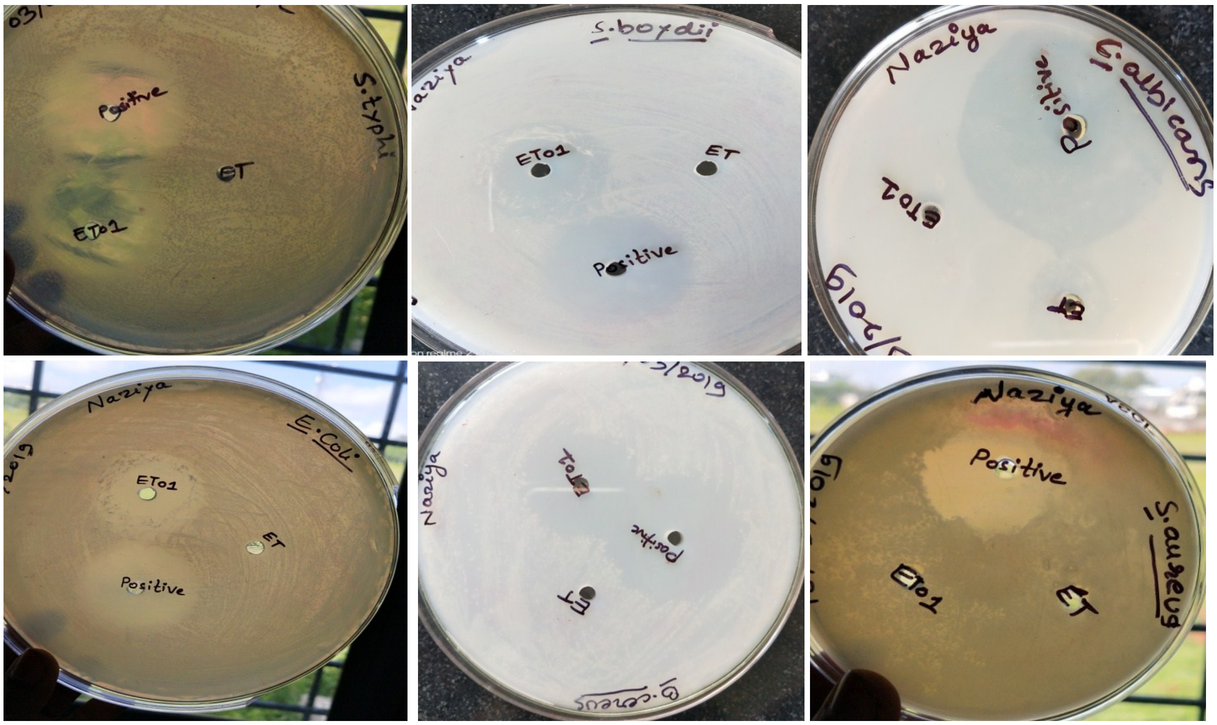

Table.1. Comparison of determination ETO with different methods of analysis with electrochemical method.

Sr. No.	Methods	LOD (µmol/L)	Linearity range	Reference
1	Spectroscopic	110	100-10000	1
2	Fluorimetric	80	1000-8000	2
3	Ion-pair chromatography	50	0.1-3.0	3
4	UV-Spectrophotometric Method	0.076	6-18	4
5	Electrochemical	1.33	0.001-100	Present work

Table.2. The electrocatalytic determination of ETO by using different modified electrodes in literature.

Sr. No.	Electrodes/ Methods	LOD (µmol/L)	Linearity range	Reference
1	Poly(l-cysteine) film-modified glassy carbon electrode	0.531	2.38–248.0	5
2	Boron-Doped Diamond Electrode	0.294	1.00 to 80.0	6
3	Zirconia nanoparticles modified electrode	47.66	150–650	7
4	Graphene Oxide modified electrode	1.33	0.001-100	Present work

References

1. M.I. Walash, A.M. El-Brashy, M.E.S. Metwally and A.A. Abdelal, *Bull. Korean Chem. Soc*., 2004, **25**, 517-524.
2. M.I. Walash, A.M. El-Brashy, Metwally and A.A. Abdelal, *J. Chin. Chem. Soc*., 2004, **51**, 1059-1064
3. C. J. Bhanushali, A. S. Zidan, Z. Rahman, M. J. Habib, *AAPS Pharm.Sci.Tech*. 2013 **4**,1313-1320
4. S. Kumar Debnath, S. Saisivam and M. Debnath, *Int. J. Drug Dev & Res*.,2017, **9**, 20-23.
5. B. R. L. Ferraz, F. R. F. Leite, A. R. Malagutti, *Talanta*., 2016,**154**, 197-207.
6. B. R. L. Ferraz, F. R. F. Leite, B. L. Batista and A. R. Malagutti, *J. Braz. Chem. Soc*., 2016, **27**, 677-684.
7. R. Jain, D. C. Tiwari, P. Pandey, *J Mol Liq*., 2014,**198**, 364-368.
